# Dopamine Genes (DRD2/ANKK1-TaqA1 and DRD4-7R) and Executive Function: Their Interaction with Obesity

**DOI:** 10.1371/journal.pone.0041482

**Published:** 2012-07-25

**Authors:** Mar Ariza, Maite Garolera, Maria Angeles Jurado, Isabel Garcia-Garcia, Imma Hernan, Consuelo Sánchez-Garre, Maria Vernet-Vernet, Maria Jose Sender-Palacios, Idoia Marques-Iturria, Roser Pueyo, Barbara Segura, Ana Narberhaus

**Affiliations:** 1 Department of Psychiatry and Clinical Psychobiology, University of Barcelona, Barcelona, Spain; 2 Institute for Brain, Cognition and Behaviour (IR3C), Barcelona, Spain; 3 Grup de Recerca Consolidat en Neuropsicologia (SGR0941), Barcelona, Spain; 4 Neuropsychology Unit, Deparment of Psychiatry, Hospital de Terrassa-Consorci Sanitari de Terrassa, Terrasa, Spain; 5 Molecular Genetics Unit, Hospital de Terrassa-Consorci Sanitari de Terrassa, Terrasa, Spain; 6 Pediatric Endocrinology Unit, Department of Pediatrics, Hospital de Terrassa-Consorci Sanitari de Terrassa, Terrassa, Spain; 7 CAP Terrassa Nord, Consorci Sanitari de Terrassa, Terrassa, Spain; University of Granada, Spain

## Abstract

Obesity is a multifactorial disease caused by the interaction between genotype and environment, and it is considered to be a type of addictive alteration. The A1 allele of the DRD2/ANKK1-TaqIA gene has been associated with addictive disorders, with obesity and with the performance in executive functions. The 7 repeat allele of the DRD4 gene has likewise been associated with the performance in executive functions, as well as with addictive behaviors and impulsivity. Participants were included in the obesity group (N = 42) if their body mass index (BMI) was equal to or above 30, and in the lean group (N = 42) if their BMI was below 25. The DRD2/ANKK1-TaqIA and DRD4 VNTR polymorphisms were obtained. All subjects underwent neuropsychological assessment. Eating behavior traits were evaluated. The ‘DRD2/ANKK1-TaqIA A1-allele status’ had a significant effect on almost all the executive variables, but no significant ‘DRD4 7R-allele status’ effects were observed for any of the executive variables analyzed. There was a significant ‘group’ x ‘DRD2/ANKK1-TaqIA A1-allele status’ interaction effect on LN and ‘group’ x ‘DRD4 7R-allele status’ interaction effect on TMT B-A score. Being obese and a carrier of the A1 allele of DRD2/ANKK1-TaqIA or the 7R allele of DRD4 VNTR polymorphisms could confer a weakness as regards the performance of executive functions.

## Introduction

Obesity is a chronic multifactorial disease caused by the interaction between genotype and environment, and it is considered the second cause of premature and avoidable mortality, after tobacco [1], [2]. It has also been suggested as a possible risk factor for neurodegenerative diseases [3], [4] and has been associated with an increased rate of psychiatric disease, such as depression [5]. At the same time, obesity can be considered as a type of addictive disorder involving an alteration of normal cerebral functioning, one that is characterized by compulsive food intake and an inability to limit ingestion [6]. Both palatable food and drugs appear to activate the same mesolimbic dopamine reward system in the human brain and animal models [7]–[9]. Thus, dopamine genes may influence the relationship between obesity, the sensitivity to reinforcement and decision making.

DRD2 is a gene encoding the D2 dopamine receptor, which is mainly expressed in the striatum [10]–[13]. The DRD2/ANKK1-TaqIA polymorphism is located, 10 kb downstream from the DRD2 gene, in the exon 8 of the adjacent gene, denoted the ankyrin repeat and kinase domain containing 1 (ANKK1) [14]. The DRD2/ANKK1-TaqIA polymorphism modulates the density of D2 receptors. Carriers of the A1 allele have shown up to 30% reduced D2 receptor density compared to homozygous A2 allele carriers [11], [13], [15], [16]. This reduction is particularly prominent in ventral parts of the caudate and putamen. Reduced glucose metabolism is also observed in carriers of the A1 allele, not only in the striatum but also in remote areas such as the ventral and medial prefrontal cortex [17].

The A1 allele of the DRD2/ANKK1-TaqIA gene has been associated with both addictive disorders [18]–[20] and obesity [21], [22]. The A1 allelic prevalence has been reported to be significantly higher in obese individuals than in lean controls [23]–[25]. Moreover, obese subjects, relative to lean ones, have fewer D2 receptors in the striatum [22], [26], [27].

Further to these findings, imaging studies have reported that the reductions in D2 receptors are associated with decreased metabolism in prefrontal cortical regions in obese subjects [28], in whom an inverse relationship between body mass index (BMI) and D2 receptors has also been described; specifically, those individuals with the lowest D2 values had the largest BMI [22]. Lower prefrontal metabolic activity has also been reported in healthy adults with the highest BMI [28], [29].

**Table 1:** Demographic and clinic characteristics in obese subjects.

	Age	Sex/Ethnia	Weight (Kg)	Height (m)	BMI (kg/m^2^)	SAP	DAP	TGL	Total CHO	Gly
1	32	F/C	87.00	1.61	33.56	96	56.50	0.64	3.93	4.25
2	30	F/H	137.00	1.62	52.20	119	73	2.22	4.89	4.94
3	37	F/C	83.50	1.64	31.05	101.5	73	0.91	4.02	4.47
4	29	M/C	141.60	1.83	42.28	127	74.5	1.40	5.17	4.86
5	36	F/C	127.00	1.59	50.24	117	71	1.83	4.27	6.19
6	37	F/C	122.80	1.65	45.11	110	74.5	1.04	3.77	4.47
7	36	M/C	96.60	1.67	34.64	101	61	1.09	4.08	4.79
8	39	F/C	80.00	1.60	31.25	126	84	1.21	4.65	5.29
9	33	F/C	104.50	1.68	37.03	113	81.5	0.94	3.49	4.67
10	21	F/C	106.00	1.60	41.41	123	80	1.91	4.68	4.00
11	37	F/C	91.50	1.69	32.04	102	73	1.27	5.04	4.56
12	34	M/C	117.00	1.80	36.11	120	80	1.50	5.51	4.74
13	39	M/H	96.00	1.62	36.58	128	53	1.25	5.08	4.82
14	34	F/C	79.50	1.54	33.52	109.5	69.5	0.89	3.83	4.05
15	37	F/C	83.00	1.65	30.49	99.5	61.5	1.48	4.54	4.78
16	39	M/C	118.70	1.67	42.56	127	82	.48	5.34	5.08
17	35	F/C	79.40	1.62	30.25	110.5	65	0.85	3.68	4.18
18	36	F/C	94.00	1.57	38.14	120	69	0.97	4.45	4.94
19	38	M/C	115.50	1.81	35.26	115	73	1.59	4.39	4.40
20	26	M/C	147.00	1.72	49.69	111	72	1.33	3.76	5.09
21	30	F/C	113.00	1.78	35.66	112	72	1.12	4.22	4.02
22	38	F/C	92.40	1.54	38.96	104.5	60	2.27	4.98	6.70
23	25	M/C	194.00	1.72	65.58	129	70	2.64	4.58	4.66
24	36	F/C	100.00	1.62	38.10	110	80	.65	4.34	5.15
25	21	F/C	142.50	1.71	48.73	120	69	1.57	3.92	5.67
26	24	F/C	98.00	1.68	34.72	106.5	66.5	0.99	3.61	4.10
27	19	F/C	129.00	1.69	45.17	111	73	1.25	4.54	4.68
28	35	F/C	87.00	1.61	33.56	100	68	0.49	4.53	4.40
29	20	F/C	118.00	1.68	41.81	115	80	0.61	4.06	4.62
30	22	F/C	84.00	1.61	32.43	115	71.5	1.15	4.06	4.34
31	27	M/C	85.00	1.68	30.10	111	78	1.55	4.79	4.24
32	35	F/C	76.00	1.55	31.63	123.5	80	1.50	4.63	4.57
33	38	M/C	105.50	1.78	33.30	138	81	2.06	5.93	4.34
34	33	M/C	97.00	1.71	33.17	134.5	87	1.34	4.76	4.79
35	37	F/C	112.80	1.73	37.69	132.5	85	1.12	4.61	4.12
36	37	F/C	127.50	1.62	48.58	136	90	1.32	5.23	4.53
37	30	M/H	129.00	1.78	40.71	131	81.	2.57	7.16	4.90
38	35	M/C	138.00	1.77	44.05	130	80	1.56	4.52	4.20
39	31	M/C	100.00	1.66	36.29	136.5	87.5	1.69	4.18	4.77
40	30	F/C	104.50	1.75	34.12	133	82.5	2.01	4.00	5.65
41	35	F/C	87.00	1.62	33.15	131.5	75.5	1.16	4.42	4.36
42	34	M/C	109.00	1.69	38.16	135	86	2.85	5.35	5.36

C = Caucasian; H = Hispanic; SAP = Systolic arterial pressure; DAP = Diastolic arterial pressure; TGL = blood triglycerides (mmol/l); CHO = blood cholesterol (mmol/l); Gly = Glycemia (mmol/l).

There are controversial results regarding the relationship between DRD2/ANKK1-TaqIA polymorphisms and cognitive processes. Berman and Noble [30] reported significantly reduced visuo-spatial performance in healthy children with the A1 allele compared with A1 non-carriers. An association between possession of the A1 allele of the DRD2/ANKK1-TaqIA polymorphism and intelligence has also been reported, but in the opposite direction. Carriers of the A1/A1 genotype in an adult female sample had a significantly higher IQ than did carriers of the A2/A2 genotype [31]. In a sample of memory-impaired subjects, those homozygous for the A2 allele exhibited worse performance in verbal memory and general cognitive ability than did those subjects bearing the A1 allele [32].

**Table 2:** Demographic and clinic characteristics in normal-weight subjects.

	Age	Sex/Ethnia	Weight (Kg)	Height (m)	BMI (kg/m^2^)	SAP	DAP	TGL	Total CHO	Gly
1	32	F/C	44.5	1.48	20.32	116.5	66	0.65	4.47	4.42
2	25	F/C	47.5	1.63	17.88	123	76	0.53	3.68	4.98
3	27	F/C	51	1.63	19.2	104.5	64	0.7	4.05	4.37
4	30	F/C	50	1.6	19.53	110	70	0.45	3.93	4.34
5	20	F/C	56	1.62	21.34	102.5	66.5	1.01	4.87	4.22
6	22	F/H	56.9	1.6	22.23	98.5	59.5	0.78	5.24	4.69
7	37	F/C	58	1.72	19.61	125.5	77.5	0.54	3.7	4.25
8	20	M/C	80.6	1.84	23.81	120	75	0.99	4.08	5.06
9	19	F/C	52	1.64	19.33	101	61	0.48	4.27	4.38
10	19	M/C	67	1.64	24.91	100	60	0.54	4.75	4.45
11	20	F/C	62	1.64	23.05	102	67	1.05	4.56	4.43
12	19	F/C	49	1.5	21.78	132	74	0.78	3.98	4.6
13	25	M/C	65.9	1.75	21.52	114	58	0.64	3.43	4.38
14	35	F/C	56	1.61	21.6	117.5	80	0.64	4.69	4.85
15	40	M/C	68.9	1.71	23.56	100	71	1.02	4.31	4.64
16	36	F/C	54.8	1.66	19.89	95.5	64	0.89	4.39	4.44
17	35	F/C	60	1.56	24.65	131	76	0.5	4.59	4.55
18	19	F/C	63.4	1.74	20.94	108	66	0.56	3.21	4.66
19	25	M/C	78	1.84	23.04	109	65	0.66	3.82	4.29
20	20	F/C	52.5	1.54	22.14	108	67.5	1.51	4.36	4.51
21	33	F/C	50	1.64	18.59	106	72	1.12	3.77	4.22
22	33	F/C	59	1.54	24.88	99.5	63.5	1.87	4.65	4.76
23	31	M/C	82	1.91	22.48	115.5	66	0.68	3.74	4.64
24	40	M/H	51.8	1.55	21.56	101	62	0.42	3.37	4.82
25	30	M/C	67.7	1.86	19.57	123	60	1.23	4.15	4.3
26	36	F/C	59	1.54	24.88	110	71	0.58	4.96	4.53
27	34	M/H	60	1.64	22.31	104.5	64	1.3	6.29	5.09
28	38	F/C	58.5	1.58	23.43	110	77	0.81	4.86	4.38
29	32	F/C	68.5	1.78	21.62	109.5	80	0.65	4.26	4.25
30	36	F/C	55.2	1.58	22.11	121	65	0.68	6.06	4.15
31	39	F/C	55.1	1.57	22.35	116	60.5	0.42	4.37	4.43
32	36	M/C	64.5	1.75	21.06	119.5	75	0.64	4.26	5.65
33	39	F/C	61.3	1.57	24.87	117	68	1.82	4.51	4.35
34	34	F/C	59.7	1.66	21.8	107.5	69	0.61	4.74	3.86
35	30	F/C	48.2	1.59	19.07	98	63	1.04	4.14	4.06
36	31	F/C	72.8	1.72	24.61	100	60	0.63	4.39	4.21
37	31	F/C	66.4	1.74	21.93	118.5	69	0.9	4.96	4.68
38	24	M/C	93	1.93	24.97	114	67	0.51	4.12	4.85
39	24	M/C	79.8	1.84	23.57	120	70	0.55	3.89	5.04
40	39	F/C	62	1.6	24.23	110	69	0.46	4.25	5.13
41	30	M/C	68.5	1.69	23.95	100	68	0.76	5.23	4.87
42	21	F/C	69.5	1.75	22.69	117	86	0.99	4.46	4.9

C = Caucasian; H = Hispanic; SAP = Systolic arterial pressure; DAP = Diastolic arterial pressure; TGL = blood triglycerides (mmol/l); CHO = blood cholesterol (mmol/l); Gly = Glycemia (mmol/l).

**Table 3:** Comparison between obese and control subjects in demographics, neuropsychological and questionnaire scores.

	Obese (N = 42)	Lean (N = 42)	Statistic	p
			χ^2^	
Gender (F/M)	28/14	29/13	0.05	0.815
	Mean (SD)	Mean (SD)	T	
Age	31.81 (6.51)	29.67 (6.97)	1.45	0.149
Education (years)	12.26 (2.87)	13.55 (2.40)	−2.22	0.029
BMI	38.30 (7.59)	22.07 (1.97)	31.77	<0.001
HADS§	1.43 (1.71)	1.24 (1.54)	0.53	0.594
Vocabulary WAIS (scalar score)	11.26 (2.19)	11.24 (1.88)	0.05	0.958
LN§	11.79 (2.64)	11.12 (1.80)	1.35	0.179
SDMT§	54.26 (10.47)	57.21 (11.18)	−1.24	0.215
TMT B (s)	65.76 (28.89)	70.24 (24.25)	−0.76	0.444
TMT B-A (s)	36.98 (24.18)	41.90 (21.24)	−0.99	0.324
COWAT§	36.69 (10.91)	37.64 (10.98)	−0.39	0.691
Stroop interference	5.24 (6.35)	3.92 (7.98)	0.84	0.403
WCST perseverative errors§	15.36 (10.64)	15,76 (11.80)	−0.16	0.869
BITE symptoms§	9.12 (6.41)	2.71 (2.87)	5.91	<0.001
3FEQ dietary restraint§	13.66 (4.04)	10.70 (3.66)	3.51	0.001
3FEQ disinhibition§	21 (6.83)	13.57 (4.09)	6.05	<0.001
3FEQ hunger§	7.50 (2.59)	4.81 (2.37)	4.96	<0.001

§ is for raw score.

F = female; M = male; BMI =  body mass index; HADS =  Hamilton Anxiety and Depression Scale; LN =  Letters and Numbers (WAIS III); SDMT =  Symbol Digit Modalities Test; TMT =  Trail making Test; WCST =  Wisconsin Card Sorting Test; BITE =  Bulimic lnvestigatory Test Edinburgh; 3FEQ = 3-factor Eating Questionnaire.

As regards frontal functions some studies in healthy adult samples have found that carriers of the A1 allele (who have a lower density of D2 receptors) display better behavioural flexibility than do A1 non-carriers [33], [34]. Other authors have reported that A1 carriers have difficulty in learning from negative feedback in a reinforcement learning task [35] and are less efficient at learning to avoid actions that have negative consequences [36], although some investigators have failed to find an association between frontal function and the DRD2/ANKK1-TaqIA polymorphism [37].

**Table 4:** Frequencies of DRD2/ANKK1 alleles and genotypes in obese and control subjects.

	N (%)	
	Obese (N = 42)	Control (N = 42)
Allele		
A1	10 (12)	13 (15.5)
A2	74 (88)	71 (84.5)
Genotype		
A1A1	2 (4.8)	1 (2.4)
A1A2	6 (14.3)	11 (26.2)
A2A2	34 (81)	30 (71.4)

**Table 5:** Frequencies of DRD4 exon 3 VNTR alleles and genotypes in obese and control subjects.

	N (%)	
	Obese (N = 42)	Control (N = 42)
Allele (%)		
2	7 (8.3)	11 (13.1)
3	3 (3.6)	6 (7.2)
4	59 (70.2)	58 (69)
5	3 (3.6)	1 (1.2)
7	12 (14.3)	7 (8.3)
8	0	1 (1.2)
Total	84 (100)	84 (100)
Genotype (%)		
2,2	1 (2.4)	2 (4.8)
2,3	0	1 (2.4)
2,4	5 (11.9)	5 (11.9)
2,7	0	1 (2.4)
3,3	0	1 (2.4)
3,4	3 (7.1)	3 (7.1)
4,4	20 (47.6)	23 (54.8)
4,5	3 (7.1)	1 (2.4)
4,7	8 (19)	3 (7.1)
7,7	2 (4.8)	1 (2.4)
7,8	0	1 (2.4)
Total	42 (100)	42 (100)

**Table 6:** Frequencies of ‘DRD2/ANKK1-TaqIA A1-allele status’ and ‘DRD4 VNTR 7R-allele status’ in obese and control subjects.

Genotype	Obese	Control	χ^2^_df_ §	p
**DRD2/ANKK1**				
A1(+)	8 (7 F)	12 (5 F)	1.05_1_	0.22
A1(−)	34 (20 F)	30 (24 F)		
**DRD4 VNTR**				
7-R (+)	10 (4 F)	6 (3 F)	1.23_1_	0.20
7-R (−)	32 (21 F)	36 (26 F)		

The number of females in each group is given in parentheses.

§ Comparison is for the ‘allele status’ frequency.

**Table 7:** Effect of ‘group’, ‘DRD2/ANKK1-TaqIA A1-allele status’ and their interactions with executive functions and eating behavior.

	Obese		Control		F	Ef S (ή^2^)
	A1 (+) (N = 8)	A1 (−) (N = 34)	A1 (+) (N = 12)	A1 (−) (N = 30)		
	Mean (SD)/Range	Mean (SD)/Range	Mean (SD)/Range	Mean (SD)/Range		
LN§	9.87 (1.55)/7; 12	12.24 (2.65)/8; 16	11.00 (1.41)/9; 14	11.16 (1.94)/7; 15	Group = 0.010 Allele status** = **6.33 * Interaction = 4.918 *	0.00257 0.00200
SDMT§	49.12 (9.14)/36; 63	55.47 (10.51)/36; 77	53.50 (13.14)/34; 77	58.70 (10.16)/36; 77	Group = 1.72 Allele status** = **3.98 * Interaction = 0.03	0.00170
TMT B (s)	91.87 (38.59)/50; 152	59.41 (22.69)/36; 136	78.25 (25.04)/47; 123	67.03 (23.58)/37; 140	Group = 0.00 Allele status** = **8.50^**^ Interaction = 1.37	0.01107
TMT B-A (s)	56.87 (29.45)/31; 105	32.26 (20.57)/3; 107	43.58 (24.10)/21; 99	41.23 (20.38)/8; 99	Group = 0.011 Allele status** = **3.57 Interaction = 2.18	
COWAT§	34.25 (12.42)/19; 60	38.17 (10.17)/14; 66	37.50 (9.68)/18; 56	37.70 (11.61)/17; 59	Group = 0.16 Allele status** = **0.08 Interaction = 0.05	
Stroop interference	3.71 (6.28)/−2.31; 17.03	5.36 (6.34)/−5.80; 16.32	1.61 (6.67)/−13.90; 10.12	4.84 (8.36)/−21; 19	Group = 0.58 Allele status** = **1.75 Interaction = 0.13	
WCST perseverative errors§	25.75 (10.66)/12; 44	12.97 (9.15)/4; 44	18.16 (12.39)/4; 44	14.80 (11.63)/4; 48	Group = 0.98 Allele status** = **7.99^**^ Interaction = 2.72	0.03030
BITE symptoms§	14.50 (6.54)/6; 24	7.73 (5.78)/0; 24	2.75 (3.59)/0; 12	2.70 (2.58)/0; 8	Group = 45.03 ^***^ Allele status** = **5.62 * Interaction = 5.49 *	0.15788 0.01971 0.01923
3FEQ Dietary restraint§	15. 12 (5.93)/6; 23	13.27 (3.49)/6; 19	9.41 (2.99)/6; 16	11.21 (3.82)/5; 18	Group = 13.14^**^ Allele status** = **0.13 Interaction = 2.17	0.01332
3FEQ Disinhibition§	26.25 (6.38)/17; 36	19.47 (6.52)/10; 35	13. 83 (5.40)/9; 25	13.46 (3.53)/8; 24	Group = 40.90 ^***^ Allele status** = **4.40 * Interaction = 3.41	0.03970 0.00427
3FEQ Hunger§	9.50 (1.92)/7; 12	7.00 (2.52)/3; 12	5.08 (2.81)/3; 11	4.70 (2.21)/3; 12	Group = 25.934 ^***^ Allele status** = **3.403 Interaction = 1.517	0.03621

Results are controlled for depression scores.

§ raw score.

SD = Standard deviation; LN = Letters and Numbers (WAIS III); SDMT = Symbol Digit Modalities Test; TMT = Trail making Test; WCST = Wisconsin Card Sorting Test; BITE = Bulimic lnvestigatory Test Edinburgh; 3FEQ = 3-factor Eating Questionnaire.

* p<0.05; **p<0.01; ***p<0.001; Ef S = effect size: ή^2^ = 0.0099, small effect; ή^2^ = 0.0588, medium effect; ή^2^ = 0.1379, large effect.

**Figure 1:**
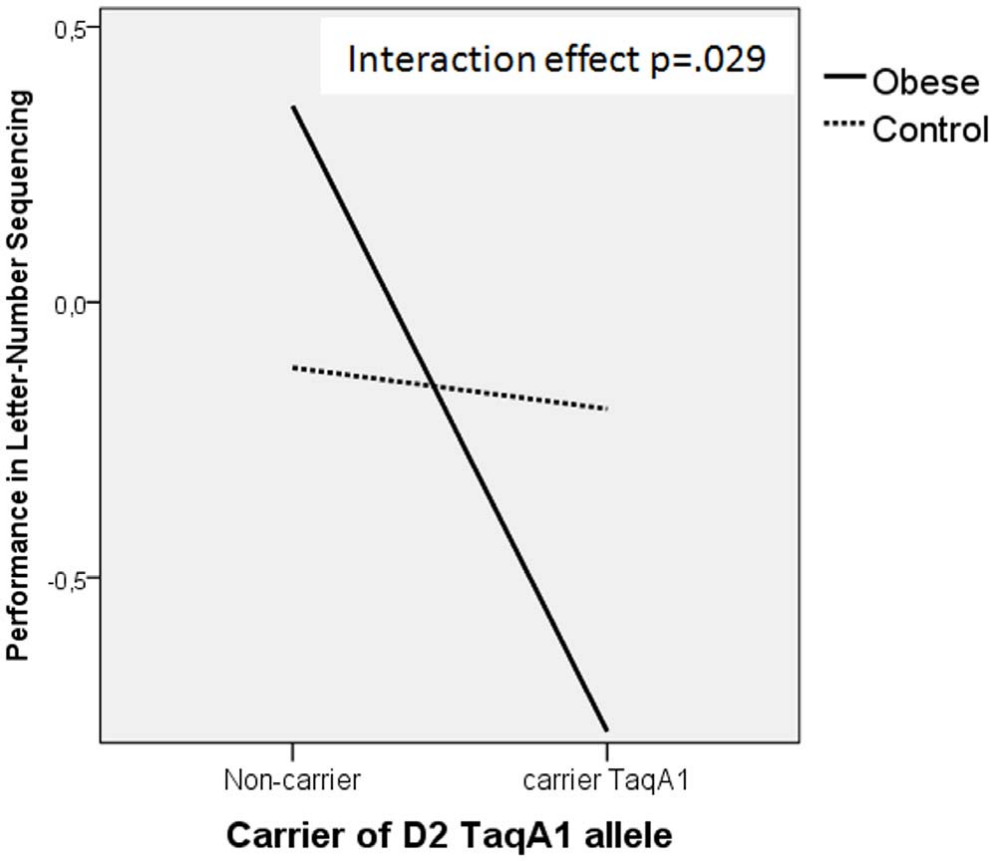
‘Group’ x ‘DRD2/ANKK1-TaqIA A1-allele status’ interaction effect on the LN score. Performance in LN is obtained by regressing depression on the dependent variable, and then saving the standardized residual from this model.

**Figure 2:**
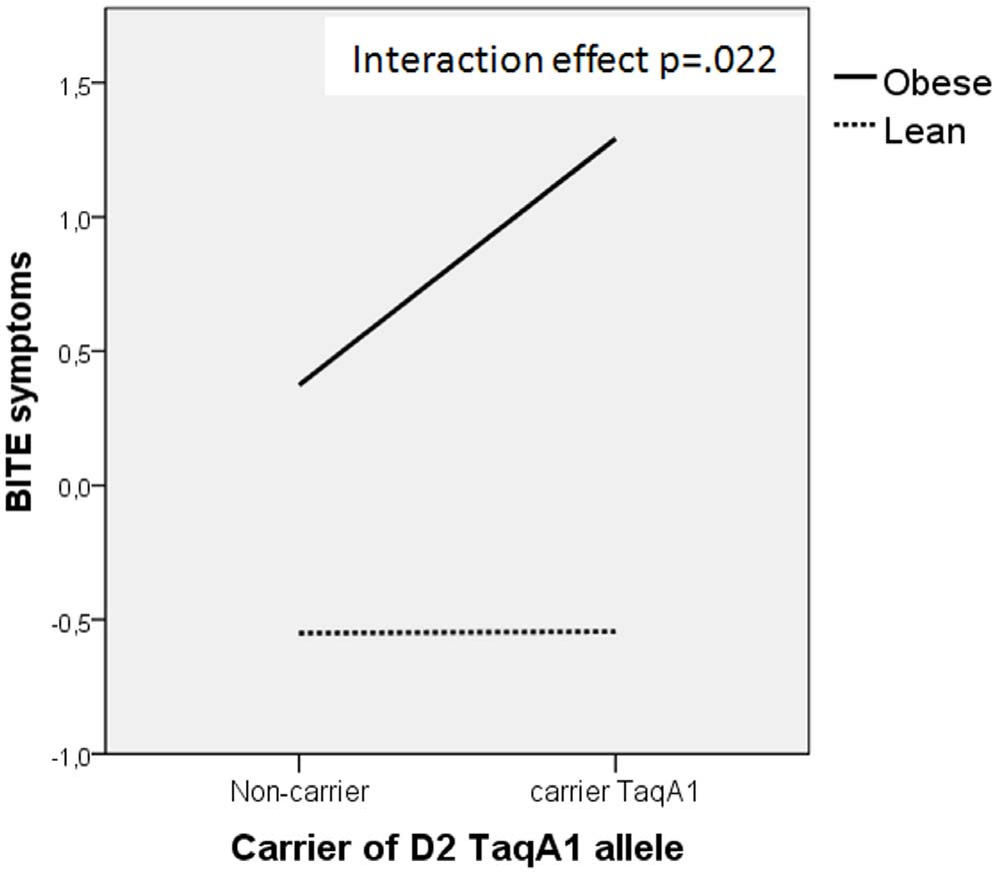
‘Group’ x ‘DRD2/ANKK1-TaqIA A1-allele status’ interaction effect on BITE symptoms. Scores in BITE symptoms are obtained by regressing depression on the dependent variable, and then saving the standardized residual from this model.

**Table 8:** Effect of ‘group’, ‘DRD4 7R-allele status’ and their interactions with executive functions and eating behavior.

	Obese		Control			F		Ef S (ή^2^)		
	7-R (+) (N = 10)	7-R (−) (N = 32)		7-R (+) (N = 6)	7-R (−) (N = 36)					
	Mean (SD)/Range	Mean (SD)/Range		Mean (SD)/Range	Mean (SD)/Range					
LN§	11.00 (2.05)/7; 13	11.65 (1.92)/8; 16		11.66 (2.33)/9; 15	11.02 (1.71)/7; 14		Group = 0.08 Allele status** = **0.08 Interaction = 1.38			
SDMT§	52.00 (11.87)/39; 77	54.96 (10.09)/36; 75		53.83 (9.84)/38; 65	57.77 (11.41)/34; 77		Group = 0.49 Allele status** = **0.03 Interaction = 0.12			
TMT B (s)	84.30 (40.87)/46; 152	59.75 (21.71)/36; 130		63.00 (16.00)/37; 84	71.44 (25.33)/41; 140		Group = 0.60 Allele status = 0.44 Interaction = 7.30^**^		0.00939	
TMT B-A (s)	51.70 (35.51)/20; 107	32.34 (17.74)/3; 89		32.50 (13.03)/18; 53	43.47 (22.05)/8; 99		Group = 0.62 Allele status = 0.12 Interaction = 9.03^**^		0.02129	
COWAT§	35.30 (5.39)/29; 44	38.09 (11.74)/14; 66		34.16 (14.53)/17; 59	38.22 (10.42)/18; 57		Group = 0.00 Allele status** = **1.07 Interaction = 0.02			
Stroop interference	4.90 (6.23)/−2.31; 15	5.10 (6.41)/−5.80; 17.03		4.60 (7.94)/−2.90; 19	3.80 (8.08)/−21; 17.60		Group = 0.21 Allele status = 0.07 Interaction = 0.07			
WCST perseverative errors§	18.70 (11.66)/7; 44	14.37 (10.24)/4; 44		17.50 (16.09)/6; 44	15.47 (11.20)/4; 48		Group = 0.00 Allele status** = **0.65 Interaction = 0.17			
BITE symptoms§	6.30 (4.47)/1; 15	9.87 (6.77)/0; 24		2.83 (3.37)/0; 8	2.69 (2.82)/0; 12		Group = 17.75 ^***^ Allele status = 1.29 Interaction = 1.09		0.06484	
3FEQ Dietary restraint§	13.55 (4.19)/8; 21	13.65 (4.06)/6; 23		10.08 (3.69)/6; 16	10.80 (3.69)/5; 18		Group = 8.93^**^ Allele status = 0.15 Interaction = 0.28		0.00937	
3FEQ Disinhibition§	18.20 (4.15)/12; 25	21.56 (7.50)/10; 36		12.66 (2.33)/10; 15	13.72 (4.31)/8; 25		Group = 20.66 ^***^ Allele status = 1.99 Interaction = 0.275		0.02075	
3FEQ Hunger§	6.60 (2.67) 3; 10	7.75 (2.55) 3; 12		4.50 (1.51) 3; 7	4.86 (2.49) 3; 12		Group = 15.22 ^***^ Allele status** = **0.96 Interaction = 0.04		0.02149	

Results are controlled for gender and depression scores.

§ raw score.

SD = Standard deviation; LN = Letters and Numbers (WAIS III); SDMT = Symbol Digit Modalities Test; TMT = Trail making Test; WCST = Wisconsin Card Sorting Test; BITE = Bulimic lnvestigatory Test Edinburgh; 3FEQ = 3-factor Eating Questionnaire.

* p<0.05; **p<0.01; ***p<0.001; Ef S = effect size: ήή^2^ = 0.0099, small effect; ήή^2^ = 0.0588, medium effect; ήή^2^ = 0.1379, large effect.

**Figure 3:**
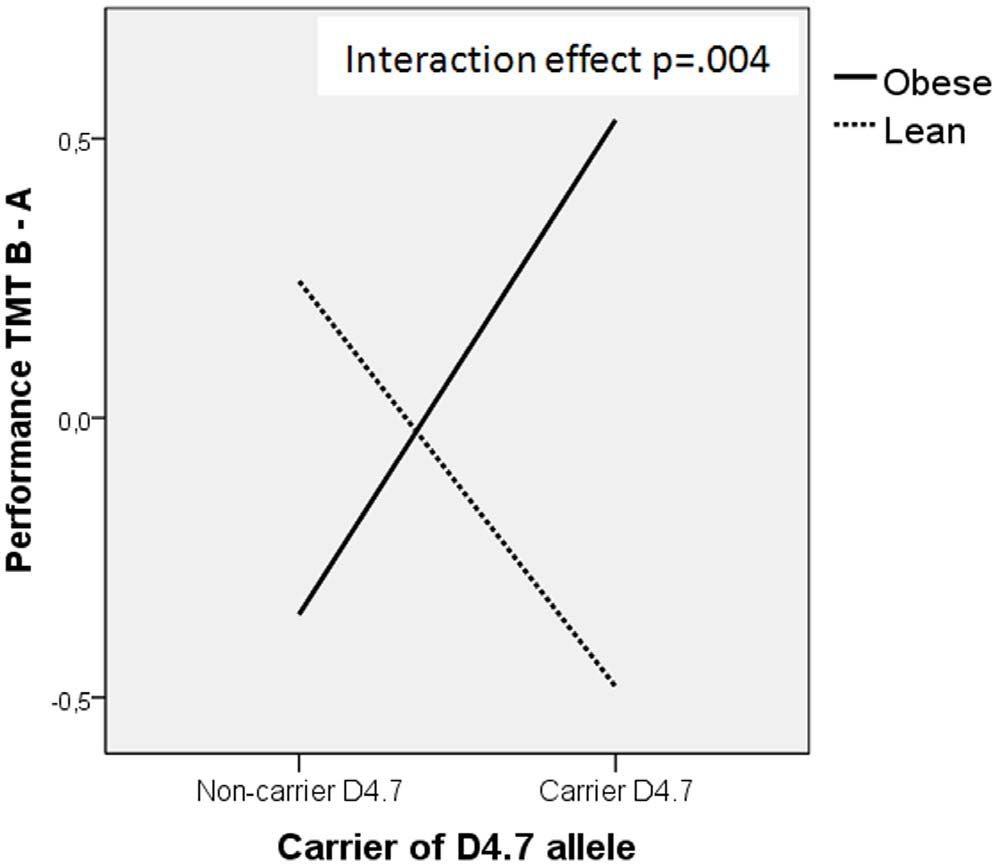
‘Group’ x ‘DRD4 VNTR 7R- allele status’ interaction effect on TMT B-A score. Performance in TMT B-A is obtained by regressing depression and gender on the dependent variable, and then saving the standardized residual from this model.

Another gene encoding dopamine receptors is DRD4, which is highly expressed in the prefrontal cortex and other brain regions that are involved in the reward circuits that mediate the reinforcing properties of food, such as the hippocampus, amygdala and hypothalamus [38], [39]. The most widely studied polymorphism of the DRD4 gene is located in the third exon and contains a 48 base-pair, variable number of tandem repeats (48-bp VNTR). Nine alleles of the DRD4 48-bp VNTR have been identified worldwide, with the number of repeats ranging between 2 and 10. The 4- and 7-repeat (7R and 4R) alleles are the most common globally [40]. The number of 48-bp repeats has been hypothesized to affect the transmitted signal in the postsynaptic neuron. Individuals with at least one allele containing seven or more repeats show both reduced binding affinities and receptor densities for dopamine neurotransmission [41].

The DRD4 gene has been associated with addictive behaviours [42] and with increased food intake in patients treated with D4 receptor-related antipsychotics [43]. The 7R allele has been linked to attention deficit and hyperactivity disorder (ADHD) [44]–[46] and to body weight gain in patients with seasonal affective disorder [47]. The 7R allele has also been found to be associated with impulsivity and lower levels of response inhibition in healthy adults, both alone and in combination with other polymorphisms in dopamine system genes, such as DRD2 [48], [49].

Research into the link between DRD4 and cognitive functions has also yielded mixed results. Some studies have reported associations between being a carrier of 7R allele and impairment on executive function tasks in healthy children [50], as well as in children with ADHD [50]–[52]. However, Manor et al. [53] reported just the opposite: their ADHD children with the DRD4 long repeats (6–8) performed better on attention than did those bearing the DRD4 short repeats (2–5). In a recent study of healthy adults, 7R allele carriers showed impaired reaction times compared with non-carriers [54]. In addition, a recent experimental study demonstrated impaired attentional performance in animals with a genetically-induced reduction in DRD4 expression [55]. Other studies, however, have shown no differences in performance on different attentional domains for 7R carriers among ADHD patients [44], [51], [53], .

Since the DRD2 and DRD4 genes have been associated with susceptibility to obesity and anomalous eating behaviour, and given that polymorphisms of these genes seem to affect the performance of executive functions, which appear to be altered in obesity, it would seem logical to ask whether there is any relationship between these polymorphisms and executive function in obese subjects. No study to date has examined a possible effect of DRD2/ANKK1-TaqIA and DRD4 VNTR polymorphisms on executive performance in the obese population. However, it must be taken into account that depression is the most frequent co-morbidity of obesity [5] and is also associated with alteration of executive functions [57]. What is more, depression has been shown to be a mediator between executive function and obesity [58], and striatal dopamine levels are low in depressive subjects [59]. For these reasons we should consider the levels of depression as a control variable in the analysis of any relationship between these polymorphisms and executive function in obese subjects.

Thus, the purpose of the present study was to assess the association between two dopamine genes and executive function and their potential interactions with obesity. The relationship between these polymorphisms and eating behaviour variables was also studied.

## Materials and Methods

Participants were randomly selected from a population base of 1539 people living within the catchment area of three public medical centres belonging to the Consorci Sanitari de Terrassa. The recruitment process continued until two samples with similar demographic features were obtained. Specifically, 816 potential participants were contacted by telephone (see the Figure S1 for detailed information on data inclusion) and 126 agreed to participate in the study; of these, 16 individuals were excluded (see criteria below) and 26 subsequently decided to leave the study. The final sample therefore comprised 84 people, with an age range of 19 to 40 years. Participants were included in the obesity group (N = 42) if their body mass index (BMI) was equal to or above 30, and in the lean group (N = 42) if their BMI was below 25. Exclusion criteria were: a history of neurological or psychiatric disorder, with the possible presence of anxiety or depression being assessed via the Hospital Anxiety and Depression Scale (HADS) [60], and the pathological use of alcohol and/or drugs being evaluated with the Structured Clinical Interview for DSM-IV (SCID-I) [61]; a history of any disorder that could be related to obesity (e.g. thyroid dysfunctions); the presence of diabetes or hypertension; BMI in the overweight range (i.e. 25–29.9); and the presence of cognitive impairment. The study was approved by the institutional ethics committee (Comissió de Bioètica de la Universitat de Barcelona (CBUB); Institutional Review Board IRB 00003099 Assurance number: FWA00004225; http://www.ub.edu/recerca/comissiobioetica.htm) and the research was conducted in accordance with the Helsinki Declaration. Written informed consent was obtained from each participant prior to taking part in the study. The demographic and clinical characteristics of the obese and control groups are shown in table 1 and table 2.

### Genotyping

Venous blood was drawn from all subjects and genomic DNA was extracted automatically by the MagNaPure Compact Instrument (Roche Applied Science, Barcelona, Spain) according to the manufacturer’s protocol. The DRD2/ANKK1 TaqIA (rs1800497) and DRD4 VNTR (rs1805186) polymorphisms were analysed as described below.

The DRD2/ANKK1-TaqIA polymorphism was amplified by polymerase chain reaction (PCR) containing 100 ng of genomic DNA from each subject, 0.5 µM each of forward (5′-GGCTGGCCAAGTTGTCTA-3′) and reverse (5′-CCTTCCTGAGTGTCATCA-3′) primers, 1× PCR buffer, 1 mM MgCl2, 200 µM of dNTPs and 2.5 units of BioTaq DNA polymerase (Bioline, Ecogen, Barcelona, Spain). The PCR programme was 95°C for 5 min followed by 35 cycles of 94°C for 30 s, 59°C for 30 s and 72°C for 1 min. The 302 bp PCR product was digested with TaqαI enzyme (New England Biolabs, Izasa, Barcelona, Spain) for 90 min at 65°C and visualized under ultraviolet light on an 8% ethidium bromide acrylamide gel. The DRD2-A2 allele was cleaved into two fragments of 176 and 126 bp, whereas the DRD2-A1 allele was not cleaved.

The DRD4 48-bp VNTR polymorphism was amplified by PCR containing 100 ng genomic DNA, 0.5 µM of each primer (5′-GCGACTACGTGGTCTACTCG-3′ as forward and 5′-AGGACCCTCATGGCCTTG-3′ as reverse), 1× PCR buffer, 1 mM MgCl2, 5% DMSO, 200 µM of dATP, dCTP and dTTP, 140 µM of dGTP, 60 µM of 7-deazaGTP and 2.5 units of BioTaq DNA polymerase (Bioline). Cycling conditions were 35 cycles at 94°C for 30 s, 63°C for 30 s and 72°C for 1 min. Amplification products were resolved by 5% acrylamide gel electrophoresis and were subsequently stained with ethidium bromide and visualized under ultraviolet light. Fragment sizes were determined by comparison with molecular length standards. A 4-repeat PCR product has a length of 475 bp. Genotyping was successful in all participants. Each genotype was recoded as carrier versus non-carrier of the allele of interest.

### Neuropsychological Assessment

All subjects underwent neuropsychological assessment of executive functions, this being based on the following tests. Letter-Number Sequencing (LN) was used to assess processing speed and verbal and visuo-spatial working memory (WM) [62], [63]. Visual scanning, tracking and motor speed were assessed by the written part of Symbol Digit Modalities Test (SDMT) [64]. Parts A and B of the Trail Making Test (TMT) were administered to measure visual scanning, motor speed and attention and mental flexibility [65]; a difference score (B-A) that removes the speed element from the test evaluation was calculated here [66]. The Controlled Oral Word Association Test (COWAT) was used to evaluate verbal fluency [67]. This measure has been shown to be sensitive to aspects of executive functioning such as initiation and sustained effort. Here, the number of words beginning with the letters F, A and S which were recalled in 1 minute was recorded. The Stroop test consists of three subtests: words, colours and colour words that conflict with the colour in which they are presented. Here the interference score was calculated as a measure of cognitive inhibitory control [68]. The Wisconsin Card Sorting Test (WCST) is a test of executive functioning that assesses cognitive flexibility and set-shifting. The individual is asked to match pictured designs based on rules that are not presented directly but which must be inferred from feedback given about the correctness of the matches; after a series of correct matches the underlying rule is changed. Here the computerized 128-card version of the WCST was used and the number of perseverative errors was recorded [69].

Eating behaviour traits were assessed using the Three-Factor Eating Questionnaire (3FEQ) [70] and the Bulimic Investigatory Test, Edinburgh (BITE) [71]. The 3FEQ is a 51-item validated questionnaire that assesses three factors (in the form of specific subscales) that refer to cognitions and behaviours: dietary restraint, disinhibition and hunger. Dietary restraint involves the conscious control of food intake in order to control body weight. Dietary disinhibition is characterized by an overconsumption of foods in response to a variety of stimuli (e.g. emotional stress) and is associated with a loss of control over food intake. Hunger represents food intake in response to feelings and perceptions of hunger. The BITE is a 33-item, self-report questionnaire designed as an objective screening test to identify subjects with bulimic symptoms and it consists of two subscales: the symptoms scale (30 items), which determines the seriousness of the symptoms, and the severity scale (3 items), which offers a severity index. The score used in the present study was that obtained from the symptoms scale, which comprises items relating to symptoms such as bulimic behaviours and weight control strategies.

### Statistical Analysis

Statistical analyses were performed by means of PASW Statistics 18.0 (SPSS Inc, Chicago, IL, USA). The Kolmogorov-Smirnov test was used to test whether all continuous variables followed a normal distribution. Frequencies of polymorphisms and alleles were calculated. Chi-square analysis tested for goodness-of-fit to the Hardy-Weinberg equilibrium. The Mann-Whitney U test was used to compare categorical variables between the genetic groups, whereas the continuous variables were compared via the Student’s t-test for independent samples. Factorial ANCOVA was performed to determine the effect of group (obesity), allele status (carrier vs. non carrier) and their interaction on executive function and eating behaviour variables controlling for confounding variables (score in HADS depression questionnaire and sex). Given the low number of participants who were simultaneously carriers of A1 (DRD2/ANKK1-TaqIA) and 7R (DRD4 VNTR), we did not examine the interaction between these two genes; instead, separate ANCOVAs were performed for each allele status (carrier vs non carrier). Finally, the effect size was calculated according to the value of eta squared (ή2). The interpretation of this statistic is as follows: ή2 = 0.0099, small effect; ή2 = 0.0588, medium effect; ή2 = 0.1379, large effect [72].

## Results

Comparisons between obese and control subjects in demographics, neuropsychological and questionnaire scores are shown in table 3.

Frequencies of DRD2/ANKK1-TaqIA alleles and genotypes, and the frequencies of DRD4 VNTR alleles and genotypes in obese and control subjects are shown in table 4 and table 5 respectively. As can be seen in Table 6 the frequency of allele carriers and non-carriers did not differ between obese and control participants for either of the two genotypes.

Although age, education and estimated intelligence were equivalent for all genetic subgroups, the DRD4-7R carriers group contained significantly more females than did the group comprising non-carriers of 7R (DRD2/ANKK1-TaqIA subgroups: age: F = 0.02; p = 0.872; years of education: F = 1.15; p = 0.286; vocabulary (WAIS): F = 0.15; p = 0.693; sex: U = 584; p = 0.47; DRD4-7R subgroups: age: F = 0.02; p = 0.869; years of education: F = 0.00; p = 0.950; vocabulary (WAIS): F = 0.50; p = 0.479; sex: U = 390; p = 0.032). Therefore, to rule out the effect of sex on the DRD4 7R allele comparisons we introduced additionally this variable in the ANCOVA analysis.

Table 7 shows the effect of ‘group’, ‘DRD2/ANKK1-TaqIA A1-allele status’ and their interaction on executive functions and eating behaviour variables controlled for depression. No significant ‘group’ effects were observed for any of the executive variables. However, there was a significant main effect of ‘allele status’ on LN SDMT, TMT B, and the number of perseverative errors on the WCST. Subjects bearing the A1 allele performed worse than those non A1-carriers subjects. There was also a significant ‘group’ x ‘DRD2/ANKK1-TaqIA A1-allele status’ interaction effect on the LN score (Figure 1).

As expected, there was a significant ‘group’ effect on all the eating behaviour variables. Interestingly, however, the results also revealed a significant effect of ‘allele status’ on two of these variables (BITE symptoms and the disinhibition subscale of the 3FEQ), as well as a significant ‘group’ x ‘DRD2/ANKK1-TaqIA A1-allele status’ interaction effect on BITE symptoms (Figure 2).

Table 8 shows the effect of ‘group’, ‘DRD4 VNTR 7R-allele status’ and their interaction on executive functions and eating behaviour controlled for gender and depression scores. No significant main effects of ‘group’ or ‘allele status’ were observed for any of the executive variables. However, there was a significant ‘group’ x ‘DRD4 VNTR 7R- allele status’ interaction effect on TMT B and TMT B-A scores (Figure 3). Eating behaviour variables these were all subject to a significant main effect of ‘group’.

## Discussion

The purpose of this study was to examine the association between two dopamine genes and executive function and their potential interactions with obesity. Specifically, the two dopamine system genes chosen were the DRD2/ANKK1-TaqIA and DRD4 48 bp VNTR polymorphisms. Both have been associated with psychiatric disorders involving impulsivity and obesity. In addition, these polymorphisms appear to exert a functional influence on dopamine D2 and D4 receptors, which are densely located in the corticostriatal-mesolimbic system [10]–[13], [73], [74], and they could potentially affect performance of frontal functions via frontosubcortical circuits linking the frontal cortex to distinct areas of the striatum [75], [76].

Previous studies have demonstrated altered executive function in obese subjects [77]–[79]. By contrast, we found no relationship between obesity and worse performance on executive function variables, there being no significant effect of ‘group’ on any of the executive variables analysed. Conversely, the A1 allele of the DRD2/ANKK1-TaqIA polymorphism was clearly associated with poor performance on executive tasks in both obese and control subjects. Specifically, significant differences between the two ‘allele status’ groups were observed for four of the seven executive variables analysed, with A1 allele carriers performing worse than A1 non-carriers.

Studies of frontal function and the DRD2/ANKK1-TaqIA polymorphism in healthy adult samples have yielded conflicting results. Some recent investigations have revealed carriers of the A1 allele to have better cognitive flexibility than A1 non-carriers [33], [34]. By contrast, other authors have shown that A1 carriers are less efficient at learning to avoid actions that have negative consequences [36] and that they have difficulty in learning from negative feedback in a reinforcement learning task [35]. Our A1 allele carriers (including both obese and control subjects) showed worse performance on variables involving motor speed, verbal and visuo-spatial working memory, set-shifting, attention and tracking, and cognitive flexibility. These results are therefore in line with those studies that have shown greater difficulty in performing tasks involving executive functions in subjects carrying the A1 allele, regardless of the test/task used [35], [36].

Our results also revealed a significantly ‘group’ x ‘DRD2/ANKK1-TaqIA A1-allele status’ interaction effect on the LN variable. In the obese group, A1 carriers performed worse than did A1 non-carriers, whereas among controls the performance of A1 carriers and non-carriers was similar. This suggests that possession of the A1 allele among obese subjects is associated with worse performance on working memory, and it could therefore confer a disadvantage in terms of executive performance. Other studies with pathological samples involving the dopaminergic system have yielded discordant results regarding frontal function. In a sample of alcoholic males those subjects bearing at least one copy of the A1 allele showed lower attention and less inhibitory control than did those without the A1 allele [80], whereas Bombin et al. [81], in a sample of psychotic adolescents, failed to detect an effect of the DRD2/ANKK1-TaqIA polymorphism on executive functions.

The A1 allele of the DRD2/ANKK1-TaqIA gene has been associated with addictive disorders [18]–[20]. Significant associations between the A1 allele of the DRD2/ANKK1-TaqIA polymorphism and variables that indicate pathological food intake were found in our sample. Subjects carrying the A1 allele scored higher on the BITE scale and on the disinhibition subscale of the 3FEQ, implying greater overconsumption of foods associated with a loss of control over food intake. We also found a significant interaction between ‘group’ and ‘DRD2/ANKK1-TaqIA A1-allele status’ in relation to BITE symptoms. In the obese group, carriers of the A1 allele scored significantly higher on the BITE than did A1 non-carriers. These results were expected since food reinforcement and impulsivity have been reported to be greater in obese individuals with the A1 allele [48], [82].

No significant ‘group’ or ‘DRD4 VNTR 7R-allele status’ effects were observed for any of the executive variables analysed. In this regard, our data partially support the results of previous studies that found no differences among ADHD patients in relation to the DRD4 VNTR polymorphism and cognitive functions [44], [51], [53], [56]. Interestingly, however, we did find a significant ‘group’ x ‘DRD4 VNTR 7R-allele status’ interaction effect on TMT B and TMT B-A scores. Specifically, in the obese group the performance of 7R carriers was worse than that of 7R non-carriers, whereas among controls the performance of 7R carriers was better than that of non-carriers. These results are consistent with previous studies that have reported associations between being a carrier of 7R and impairment on executive function tasks in healthy children [50], adults [54] and in children with ADHD [50]–[52]; they also support the findings of those authors who have reported better performance among subjects with the DRD4 long (6–8) repeats compared to the DRD4 short (2–5) repeats [53]. It would seem, therefore, that obesity is a key factor in relation to possession of the allele. Being obese and having the DRD4 7-repeat allele appears to confer a weakness in terms of the performance of executive functions.

At all events the strengths and limitations of the present study should be considered when interpreting the findings. The study sample size was modest, and research designed to identify genes that are associated with specific behaviours usually requires a much larger sample. Nevertheless, this sample size did provide enough statistical power to detect a significant interaction and main effect of gene variants on our measures of executive function, even though the effect sizes were small. The results should, however, be interpreted with caution in view of the numerous statistical comparisons, the risk being that some of the findings may be spurious. The present results therefore require confirmation by studies that are adequately powered to detect the effect of DRD2-A1 and DRD4-7R on executive performance.

Ideally one would also have investigated all possible interactions among the two polymorphisms studied here. However, for the DRD4 48-bp VNTR and the DRD2/ANKK1-TaqIA polymorphisms the genotype frequencies involving the minor allele were too low to test for meaningful interactions. Future studies should therefore address the question of all potential interactions between these two (and other) polymorphisms in dopamine system genes in a larger, adult, obese cohort.

To conclude, despite the methodological limitations, we found that obese subjects carriers of the A1 allele of the DRD2/ANKK1 TaqA1 and carriers of the 7R allele of the DRD4 48-bp VNTR polymorphism performed worse on certain tests of executive functions. These results provided new knowledge on the effect of two genes that are crucial for dopamine neurotransmission, which underlies several psychiatric disorders. The study of the genes related to dopamine transmission, with respect to both its elimination and its release or receptor binding, may shed light on the role of this system in mental disorders.

## Supporting Information

Figure S1 **The figure shows detailed information on data inclusion.**
(TIF)
