# Supplementary figures and images for: Dopamine Genes (DRD2/ANKK1-TaqA1 and DRD4-7R) and Executive Function: Their Interaction with Obesity
Source: PLoS One. 2012 Jul 25;7(7):e41482. doi: 10.1371/journal.pone.0041482 (PMC3405092; doi:10.1371/journal.pone.0041482)

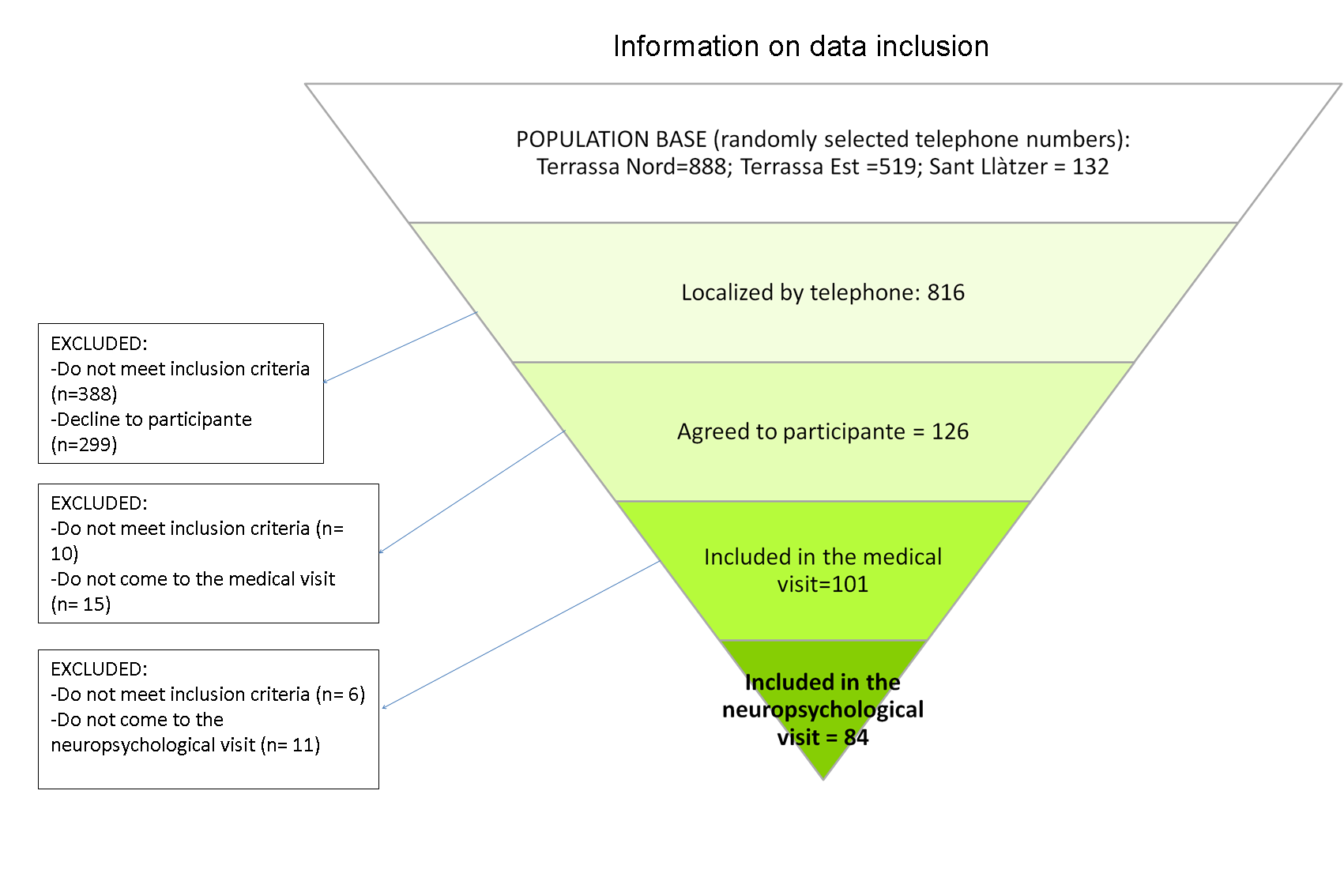

Supplement: Figure S1 — The figure shows detailed information on data inclusion. (TIF) [file pone.0041482.s001.tif]
